# A Host-Mimicking Peanut-Based Selective Medium Enhances Isolation, Sporulation, and Metabolomic Profiling of *Aspergillus flavus*

**DOI:** 10.3390/microorganisms14102391

**Published:** 2026-10-19

**Authors:** Yranney Brice Nganguem Nzalle, Yanyan Wang, Gang Luo, Xiaochuan Rang, Gongyou Zhang, Yingqian Kang

**Affiliations:** Guizhou Key Laboratory of Microbiome and Infectious Disease Prevention and Control & Key Laboratory of Environmental Pollution Monitoring and Disease Control, Ministry of Education of Guizhou & School of Basic Medical Science & Institution of One Health Research, Guizhou Medical University, Gui’an New District, Guiyang 561113, China; yranneybrice@yahoo.fr (Y.B.N.N.); wang_yanyan@gmc.edu.cn (Y.W.); luogang@gmc.edu.cn (G.L.);

**Keywords:** *Aspergillus flavus*, host-mimicking medium, selective isolation, sporulation, metabolomics, peanut-based selective medium (PSM), corn-based selective medium (CSM)

## Abstract

*Aspergillus flavus* is a significant foodborne pathogen, responsible for food contamination and invasive aspergillosis. Isolating this pathogen is challenging because traditional media do not reflect the natural environment. In this study, we designed peanut- and corn-based selective media, PSM and CSM, to improve isolation efficacy, growth, sporulation, and metabolic profiles compared with conventional media (PDA and SDA). A novel peanut- and corn-based selective medium (PSM and CSM) improved *A. flavus* recovery, radial expansion, and sporulation compared with PDA and SDA. These effects were reproducible across isolate origins, indicating that nutrient composition, not strain source, was the key driver. LC-MS/MS-based metabolomic profiling revealed strain- and substrate-specific metabolic phenotypes. Corn- and peanut-based media differentially modulated amino acid metabolism, lipid remodeling, and secondary metabolite biosynthesis. Under host-relevant conditions, the clinical isolate downregulated multiple amino acid pathways and upregulated glycerophospholipid metabolism. Altogether, PSM provides a powerful platform to isolate and functionally evaluate *A. flavus*, improving phenotypic resolution and revealing biologically relevant metabolic differences hidden in traditional culture. This has implications for clinical mycology, food safety, and mycotoxin studies. These results are exploratory and hypothesis-driven, as the clinical metabolic phenotype was defined in a single clinical isolate, and need to be confirmed on a more diverse and geographically expanded clinical isolate collection before generalization.

## 1. Introduction

*Aspergillus flavus* belongs to the phylum Ascomycota and the family Trichocomaceae and is highly significant in both agriculture and human health [1,2]. It is the main cause of invasive aspergillosis predominantly in immunocompromised people [3,4,5]. In addition, *A. flavus* contaminates staple foods such as peanuts, maize, and tree nuts [6] by producing aflatoxins, particularly aflatoxin B1 (AFB1), one of the most potent naturally occurring hepatocarcinogens [7,8,9]. Therefore, accurate diagnosis, identification, and characterization of *A. flavus* strains are paramount for public health and food safety [10,11].

*A. flavus* is generally studied in the laboratory using standard culture media that include Potato Dextrose Agar (PDA) and Sabouraud Dextrose Agar (SDA) [12]. Although these media support robust mycelial growth, they also promote contamination, reducing *A. flavus* recovery when analyzing complex samples [13].

In addition, their nutritional profiles differ significantly from the multifaceted systems found in natural host substrates [14]. The ecophysiology of *A. flavus* indicates that toxigenic capability and metabolic activity can be misrepresented when grown in standard carbon-rich culture media; mycotoxin production may be especially environment- and nutrient-specific [15,16]. Selective culture media better reflect the nutritional and environmental conditions during colonization [16]. Peanuts (Arachis hypogaea) are a major source of fungal infection and provide proteins, lipids, and bioactive components that may regulate fungal growth and secondary metabolite biosynthesis [17]. Corn is another favored environmental niche where *A. flavus* can grow [18].

Incorporating these substrates into selective media not only meets nutritional needs but also supports phenotypic activities, including growth and sporulation, under laboratory conditions that more closely match the natural environment [19]. Beyond substrate composition, selective isolation can be further improved by altering pH and osmotic pressure and adding antibiotics or other inhibitory compounds to suppress other microorganisms [20]. Rose Bengal, a visual differentiation agent, can help distinguish *A. flavus* from other fungi [21].

A host-mimicking substrate combined with physicochemical and antimicrobial selection is a logical method for improving recovery efficiency, colony selection, and sporulation [22]. Despite these advantages, no systematic study of *A. flavus* has used peanut- or corn-based media. Previous comparative studies often focus on limited parameters, such as radial growth and secondary metabolites, but not sporulation [23].

To address this gap, the current study aimed to formulate a novel peanut- and corn-based medium and compare its performance with conventional lab media (PDA and SDA). We compared a variety of phenotypic and metabolic parameters of clinical and environmental *A. flavus*. The peanut-specialized medium realistically reflects natural physiology and toxigenicity and will serve as a strong platform for ecological, clinical, and food safety research.

## 2. Materials and Methods

### 2.1. Strains and Culture Conditions of Fungi

Fourteen environmental fungal isolates (HJ-1 to HJ-14) were isolated from various agro-ecological and geographical regions of China. In contrast, the fungal isolate L-1-22 was isolated from a respiratory specimen at Jining Hospital, Shandong Province, China. Isolate HJ-3 was excluded from the analysis after morphological and multi-locus molecular identification (ITS, BenA, and CaM), which showed it to be another species of *Aspergillus* other than *A. flavus*. In total, there were thirteen environmental *A. flavus* isolates (HJ-1, HJ-2 and HJ-4 to HJ-14) and one clinical *A. flavus* isolate (L-1-22). Geographic coordinates and elevation for each sampling site are listed in Supplementary Table S1. All isolates were purified by single spore culture, and conidial suspension was stored in 20% (*v*/*v*) glycerol at −80 °C and was cultured monthly on potato dextrose agar (PDA; Hopebio, Qingdao, China). All *A. flavus* isolates were confirmed using multi-locus sequencing, and the sequences of the primers and the GenBank accession numbers are listed in Supplementary Tables S2 and S3.

### 2.2. Formulation and Preparation of Selective-Enrichment Media

Two substrate-based selective-enrichment media were designed: Peanut-Based Selective Medium (PSM) and Corn-Based Selective Medium (CSM). The formulation combines various selective principles that favor *A. flavus* and suppress other microbiota. Selection for this formulation was based on the known ecophysiology of *A. flavus* and mycological literature, and not on empirical data from the present study. *Aspergillus flavus* is a significant pathogen of peanut and corn and is well adapted to utilize host proteins, lipids, and carbohydrates [24]. It is also tolerant of mildly acidic conditions, high osmotic pressure, and selective agents like Rose Bengal and dichloran at levels that may kill competing bacteria, yeasts, and fast-growing fungi [25]. Thus, three complementary selective principles were incorporated in the formulation: (i) the presence of Rose Bengal and dichloran, provided in the DRBC agar base, inhibits the growth of other molds and restricts colony spread [25]; (ii) chloramphenicol (50–100 mg L^−1^) suppresses bacterial growth [26]; (iii) the addition of sodium chloride (15–20 g L^−1^) and the acidic pH (5.8 ± 0.2) impose osmotic and physicochemical stress on sensitive microorganisms [27]. The medium has been formulated a priori to include conditions suitable for the growth of *A. flavus* and to inhibit the growth of non-target bacteria, yeasts, and filamentous fungi using host-mimicking substrates and these selective conditions. Each ingredient was selected specifically to match the nutritional needs and metabolic capabilities of *A. flavus*. Defatted peanut and corn powder were chosen as basal matrices because *A. flavus* is naturally adapted to these crops as ecological niches and possesses the enzymatic machinery to break down complex proteins, carbohydrates, and lipids for robust growth, sporulation, and secondary metabolite production [24]. To prepare the substrate extract, 50 g of defatted peanut powder or yellow corn powder was mixed with 500 mL distilled water, stirred, and heated at 60–70 °C for 30 min. The suspension was then passed through sterile cheesecloth, and the clear filtrate was then used to prepare the respective selective medium. Dextrose (20 g L^−1^) was provided as an easily assimilable carbon source to promote rapid conidial germination and biomass accumulation during early colonization [28]. The pH range (5.8 ± 0.2) and elevated sodium chloride (15–20 g L^−1^) have selective functions and mimic the osmotic and acidic microenvironment naturally present in peanut and corn tissues, to which *A. flavus* is adapted [20]. Therefore, each component was selected in light of prior knowledge of the physiology of *A. flavus* and its natural host habitat, to provide a rationally formulated medium that was consistent with the intrinsic growth requirements of the species.

### 2.3. Sample Processing and Selective Enrichment Efficiency Assessment

Nine samples were collected from different sites, including moldy nuts and grains, and were processed to isolate the fungi. One gram of each sample was homogenized in 9 mL sterile distilled water supplemented with 0.05 milliliters of Tween-80 [20]. A ten-fold dilution was prepared, and 100 µL of the dilution was spotted and spread on PSM, CSM, PDA, and SDA in triplicate. The samples were incubated at 30 °C for 5 days. Colonies were enumerated and categorized, and the selective efficiency was estimated as (Number of *A. flavus*-like colonies)/(number of total colonies) × 100. Presumptive colonies of *A. flavus* were first identified according to their morphology and subsequently verified by multi-locus sequencing (ITS, BenA, CaM). The recovery and the selectivity were calculated after molecular confirmation. Selectivity was evaluated by comparing the number of *A. flavus* colonies that were confirmed to the number of recovered colonies, and by counting distinct non-target colony morphotypes.

### 2.4. Growth Kinetics and Sporulation Assays

Radial growth was measured using the standard mycelial plug technique [29]. We collected a 5 mm agar plug from a 5-day-old culture and inoculated it at the center of each medium tested in replicates. Incubation was done at 28 °C and 30 °C. The diameter of the colonies was measured at an interval of 24 h for seven consecutive days. Sporulation was observed between days 5–7 by removing an agar plug (1–2 cm), suspending all conidia in saline-Tween, and counting them in a hemocytometer.

### 2.5. Extraction and LCMS/MS Based Metabolomic

#### 2.5.1. Metabolite Extraction

The 5-day-old cultures were used to harvest fungal biomass, washed with phosphate-buffered saline, frozen in liquid nitrogen, and lyophilized. About 20 mg of dry biomass was added to a mixer mill with zirconia beads, followed by extraction with 400 µL of ice-cold extraction solvent (methanol: acetonitrile: water, 2:2:1, *v*/*v*/*v*). The mixture was vortexed and then incubated at −20 °C and ultrasonicated in an ice bath for 30 min. The supernatant was centrifuged at 14,000× *g* for 15 min at 4 °C and dried using a centrifugal concentrator (Labconco, Kansas City, MO, USA); LC-MS analysis was performed after reconstitution in 100 µL of acetonitrile: water (1:1, *v*/*v*) [30]. The solvents were all LC-MS grade (Merck, Darmstadt, Germany).

#### 2.5.2. LC-MS/MS Analysis

Chromatographic separation was performed on a Vanquish ultra-high-performance liquid chromatography (UHPLC) system (Thermo Scientific, Waltham, MA, USA) with a Waters ACQUITY Premier HSS T3 column (1.8 µm × 0.21 mm × 100 mm) maintained at 40 °C. The mobile phase was as follows: (A) water with 0.1% formic acid and (B) acetonitrile (ACN); the gradient program is given in Supplementary Table S4. The flow rate was set to 0.4 mL min, and 3 µL was injected.

Mass spectrometric analysis was done using the Q Exactive HF-X hybrid quadrupole-Orbitrap mass spectrometer (Thermo Scientific, Waltham, MA, USA) operated in both positive and negative electrospray ionization (ESI) modes with a full scan range of m/z 70–1000. Key acquisition parameters were MS1 and MS2 at resolutions of 60,000 and 15,000, respectively; automatic gain control (AGC) set to 3 × 10^6^ and 1 × 10^5^, respectively; and stepped normalized collision energies [31]. All parameters of the mass spectrometry are explained in Supplementary Table S5. To assess system stability, we periodically injected a pooled quality control (QC) sample containing equal proportions of aliquots from all experimental samples (after every 6–8 samples) throughout the analytical sequence [32]. Independent biological replicates of fungal culture samples were used to perform the metabolomic analysis under each experimental condition. Pooled QC samples were monitored to assess technical reproducibility, instrument stability, and analytical variation during LC-MS/MS acquisition and were not considered biological replicates. Three independent biological replicates (n = 3) were analyzed for each strain/medium combination, with each replicate being cultured, harvested, and extracted independently. Only metabolites that appeared in at least two of the three replicates were included for analysis, and mean values were used for quantitative comparisons.

The input and output approach was determined by the quality control and data processing performed on the data.

#### 2.5.3. Data Processing and Quality Control

The data processing workflow was defined according to QC assessment and quality-filtering criteria. The raw data were converted to mzML format, and peaks were picked, aligned, and retention times corrected using the XCMS package (version 3.18.0) in R (version 4.3.1) [33]. Parameters with more than 50% missing values in the experimental samples were discarded. Missing values were also imputed using the k-nearest neighbors (KNN) algorithm. Data were normalized using unit variance scaling (UV scaling). To address data quality, metabolite features were screened for reproducibility in the pooled QC samples; only features with a coefficient of variation (CV) smaller than 30% in the QC group were retained for further statistical analysis [31]. The metabolites were annotated and identified using the standard metabolites database.

#### 2.5.4. Metabolite Identification and Annotation

Metabolite annotation and identification were performed using accurate mass (with a tolerance of ±10 ppm) MS/MS spectra, which were compared with standard in-house library databases (e.g., HMDB) and in silico prediction software (e.g., SIRIUS (version 6.0.4), CSI: FingerID (version 3.1)). We reported annotation confidence using Metabolomic Standards Initiative (MSI) levels. Only features with an identification confidence score of 0.5 or above and a CV of 0.3 or less in QC samples were retained for further statistical analysis.

#### 2.5.5. Statistical and Pathway Analysis

Global metabolomic profiles were assessed using unsupervised principal component analysis (PCA) with the prcomp function in R (version 4.3.1, R Foundation for Statistical Computing, Vienna, Austria). To perform pairwise comparisons of culture media, differential metabolites were detected with a fold change (FC) threshold between 0.5–2.0 and variable importance in projection (VIP) threshold of not less than 1.0 in orthogonal partial least squares discriminant analysis (OPLS-DA) models built using the ropls package [27]. Permutation tests were used to assess the robustness of OPLS-DA models and to ensure the absence of potential overfitting. We used the Complex Heatmap package (version 2.16.0) to create hierarchical clustering and heatmap visualizations [28]. Metabolite set enrichment analysis (MSEA) using the Kyoto Encyclopedia of Genes and Genomes (KEGG) database was used to perform functional pathway analysis of non-significantly differentiated metabolites. Functional pathway analysis of different metabolites was performed by metabolite set enrichment analysis (MSEA) based on the Kyoto Encyclopedia of Genes and Genomes (KEGG) database. Functional pathway analysis of different metabolites was performed by metabolite set enrichment analysis (MSEA) based on the Kyoto Encyclopedia of Genes and Genomes (KEGG) database [34].

### 2.6. Statistical Analysis

All experiments were performed with independent biological replicates. For selective-enrichment assays, three independent replicates were performed for each strain/medium combination (Section 2.3); growth kinetics assays were performed on [n] independent replicates (Table S2); and metabolomic analysis was performed on at least 3 independent biological replicates per strain/medium combination (Section 2.5.2), with metabolite features detected in at least two of the three replicates retained for mean values and used for comparison. Two-way ANOVA with Bonferroni post-hoc correction was used to analyze the radial growth data. Metabolomic data were processed in R (version 4.3.1) using XCMS (version 3.18.0), and multivariate analyses (PCA and OPLS-DA with permutation testing) were done using the ropls package. The metabolites were used to create heatmaps using the Complex Heatmap package, and pathway enrichment analysis was carried out using metabolite set enrichment analysis (MSEA) from the Kyoto Encyclopedia of Genes and Genomes (KEGG) database. Graphing software (GraphPad Prism version 10.2.0) was used to prepare figures. The *p*-value < 0.05 was considered to be statistically significant. Data are given as mean ± SEM, where applicable.

## 3. Results

### 3.1. Selective Performance of Media Based on Peanuts and Corn

**Figure 1:**
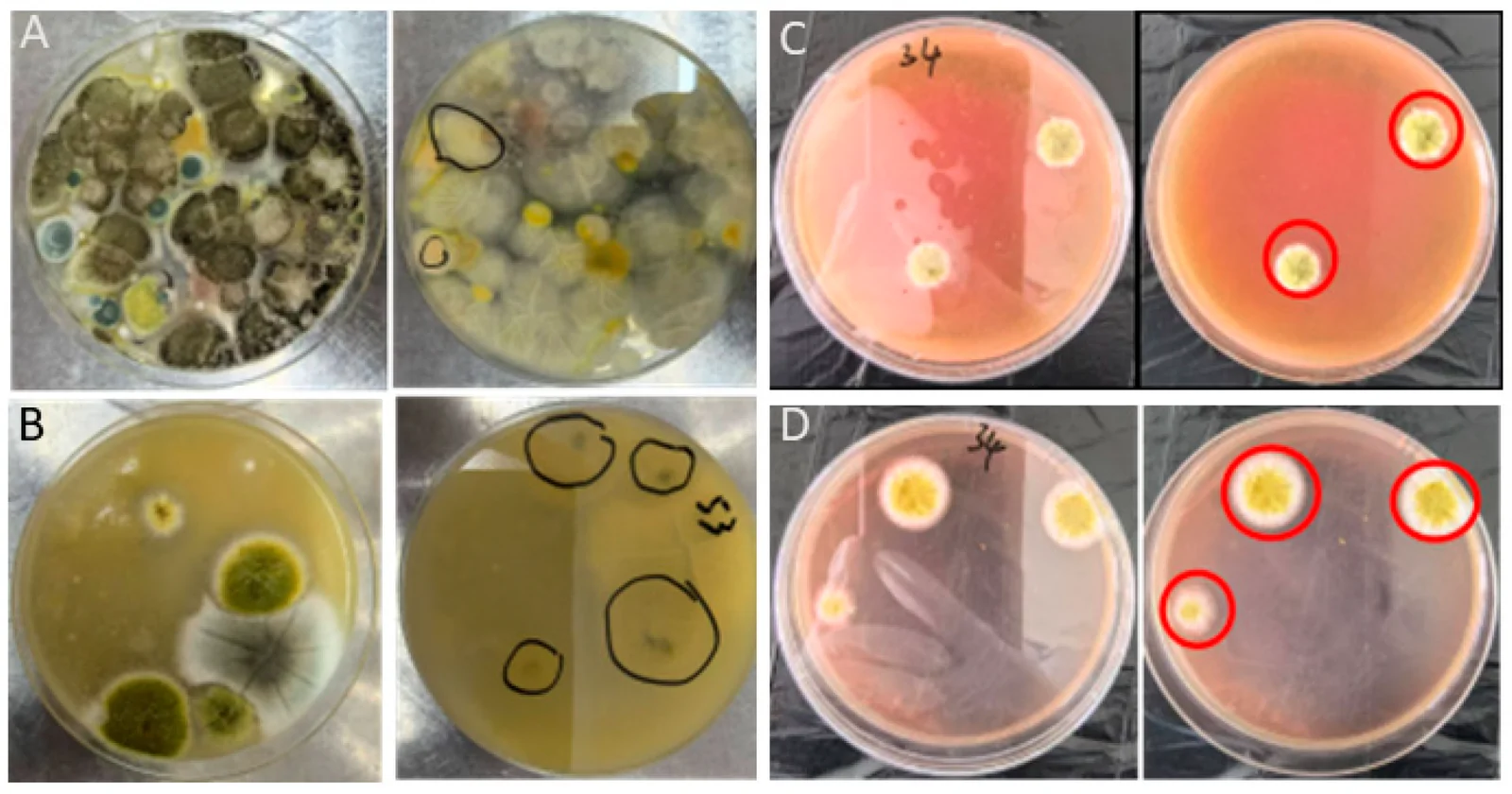
Comparative growth of *Aspergillus flavus* on various media. (**A**,**B**). Potato Dextrose Agar (PDA) & Sabouraud Dextrose Agar (SDA) with mixed growth. (**C**) Peanut-based selective medium (PSM) showed a significant decrease in background growth, with only two colonies identified. (**D**) Corn-based selective medium (CSM) with equally improved selectivity.

The effectiveness of the newly designed peanut-based selective medium (PSM) and corn-based selective medium (CSM) was evaluated by analyzing nine environmental samples and compared with traditional media, Potato Dextrose Agar (PDA) and Sabouraud Dextrose Agar (SDA). Both PSM and CSM significantly inhibited the growth of other microbes when compared to the conventional media. Only one to three non-target colony morphotypes were observed on the selective media, whereas eight to thirteen morphotypes were regularly obtained on PDA and SDA. Reducing competing microbial populations greatly improved *Aspergillus flavus* growth on PSM and CSM (Figure 1).

Between the two selective media, PSM showed greater selectivity and yielded near-pure *A. flavus* cultures in most samples. These findings highlight the importance of adding host-mimicking substrates and appropriate physicochemical selective pressures to refine selective isolation of *A. flavus* from complex environmental samples. *A. flavus* colonies on selective media reached 100%, whereas conventional media showed high mixed growth and poor differentiation, ranging from 81.81% to 85.71% (Supplementary Table S6). The initial classification was made based on the characteristic morphology of the colonies and was confirmed by microscopic examination and molecular identification.

The fourteen strains of *A. flavus* were categorized as 13 environmental strains (HJ-1, HJ-2 and HJ-4 to HJ-14) and 1 clinical strain (L-1-22). The results of molecular identification based on multi-locus sequencing (ITS, BenA and CaM) indicated a sequence similarity of 99–100% with reference strains of *A. flavus.*

### 3.2. Colony Morphology and Macroscopic Growth Features

The mean colony diameters (±SEM) of fungal strains cultured on four different media are given in Supplementary Table S7. Colony size and diameter varied across all four media, with peanut-based Selective Medium (PSM) supporting the greatest growth, followed by Corn-based Selective Medium (CSM). SDA and PDA produced the smallest colonies.

A repeated 2-way ANOVA was performed to determine the medium effect on the growth of the different fungal strains. The findings showed that the medium had a substantial effect on colony diameter (F (3, 39) = 29.41, *p* < 0.001, ηp^2^ = 0.53), validating the significant impact of medium type on fungal growth. Post-hoc pairwise comparisons with Bonferroni correction indicated a clear increase in colony diameters on PSM and CSM compared with SDA and PDA.

**Figure 2:**
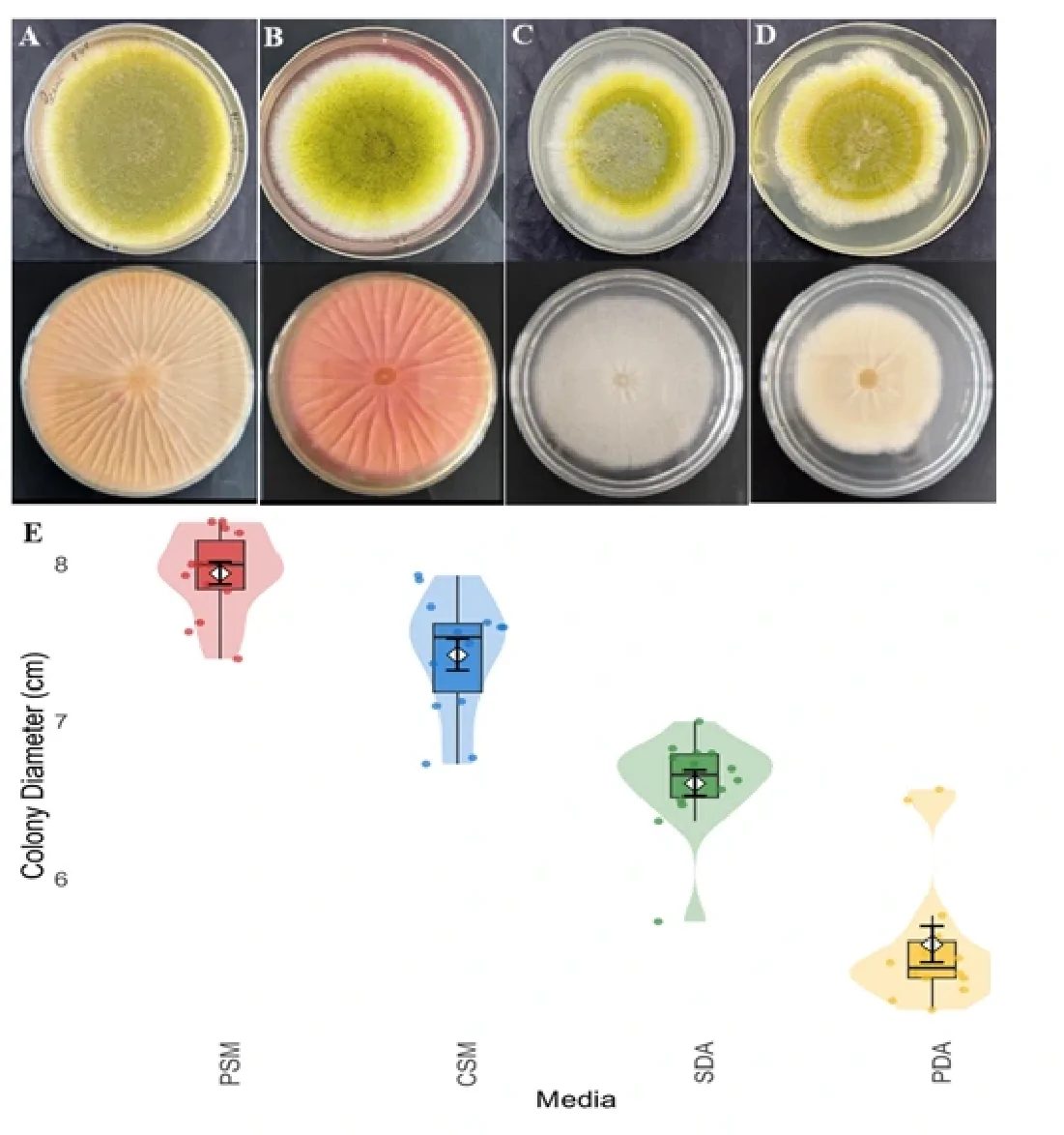
Colony morphology and radial growth of *Aspergillus flavus* on four culture media. (**A**) Peanut-Based Selective Medium (PSM); (**B**) Corn-Based Selective Medium (CSM); (**C**) Sabouraud Dextrose Agar (SDA); and (**D**) Potato Dextrose Agar (PDA). Each medium has an upper surface (obverse) and a lower reverse side (after incubation). (**E**) Violin plots displaying the distribution of colony diameter (cm) for each medium. Boxes: medians & interquartile ranges; whiskers: data spread; points: individual biological replicates.

The violin plot shows the distribution of colony diameters in each medium (Figure 2). Among these, PSM shows larger colonies than the other media. The boxplot in the middle of each violin indicates the median and interquartile range, showing central tendency and variability in each medium group. The jittered data indicate a single-strain measurement, and the error bars show the standard error of the mean (SEM) for each medium. Fungal growth differed significantly among culture media (*p* < 0.001).

### 3.3. Sporulation Efficiency in the Various Media and Temperatures

Microscopic analysis showed that sporulation capacity varied significantly among different media types. Isolates grown on PSM showed high conidiation, including thick conidiophores, well-developed conidial heads, and high spore concentration. Sporulation on CSM was intermediate, whereas PDA and SDA sustained relatively weak and unpredictable conidiation.

**Figure 3:**
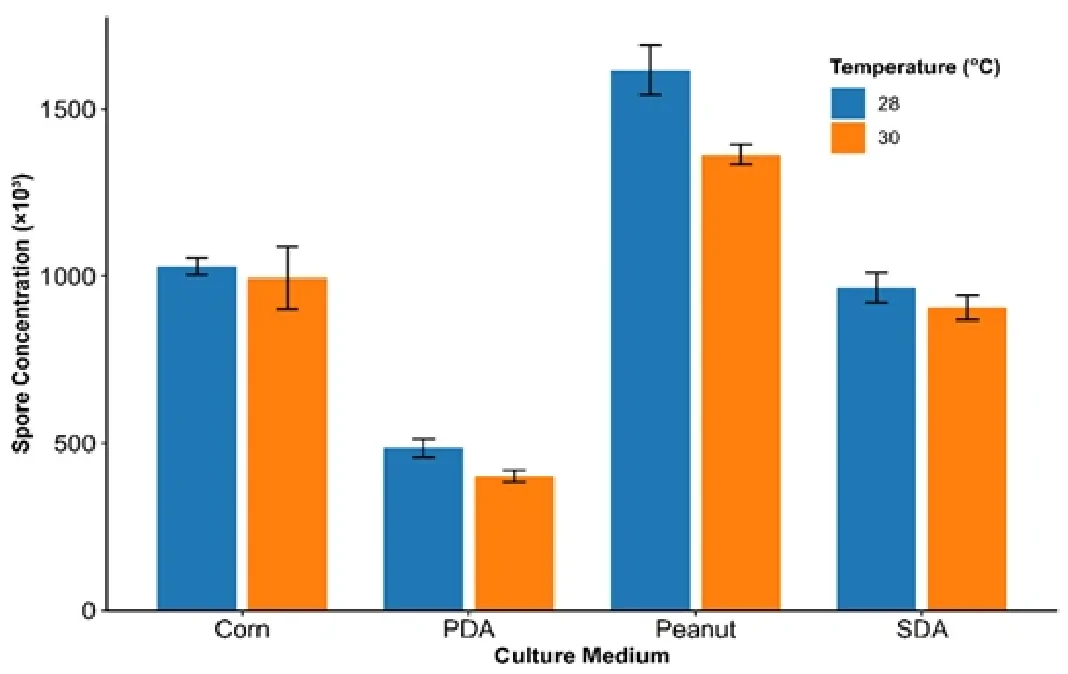
The spore concentration of Aspergillus flavus in four culture media (PDA, SDA, Corn and Peanut) at two incubation temperatures (28dºC and 30ºC). Bars indicate mean spore concentration (×103).

Incubation temperature also affected spore production. At 28 °C and 30 °C, PSM consistently supported higher spore concentrations than the other media (Figure 3). Further enrichment of sporulation on PSM is extremely beneficial for downstream uses that require high inoculum density, such as pathogenicity investigations, toxin evaluation, and environmental surveillance.

### 3.4. Microscopy, Molecular Identification and Phylogenetic Tree of Isolates

**Figure 4:**
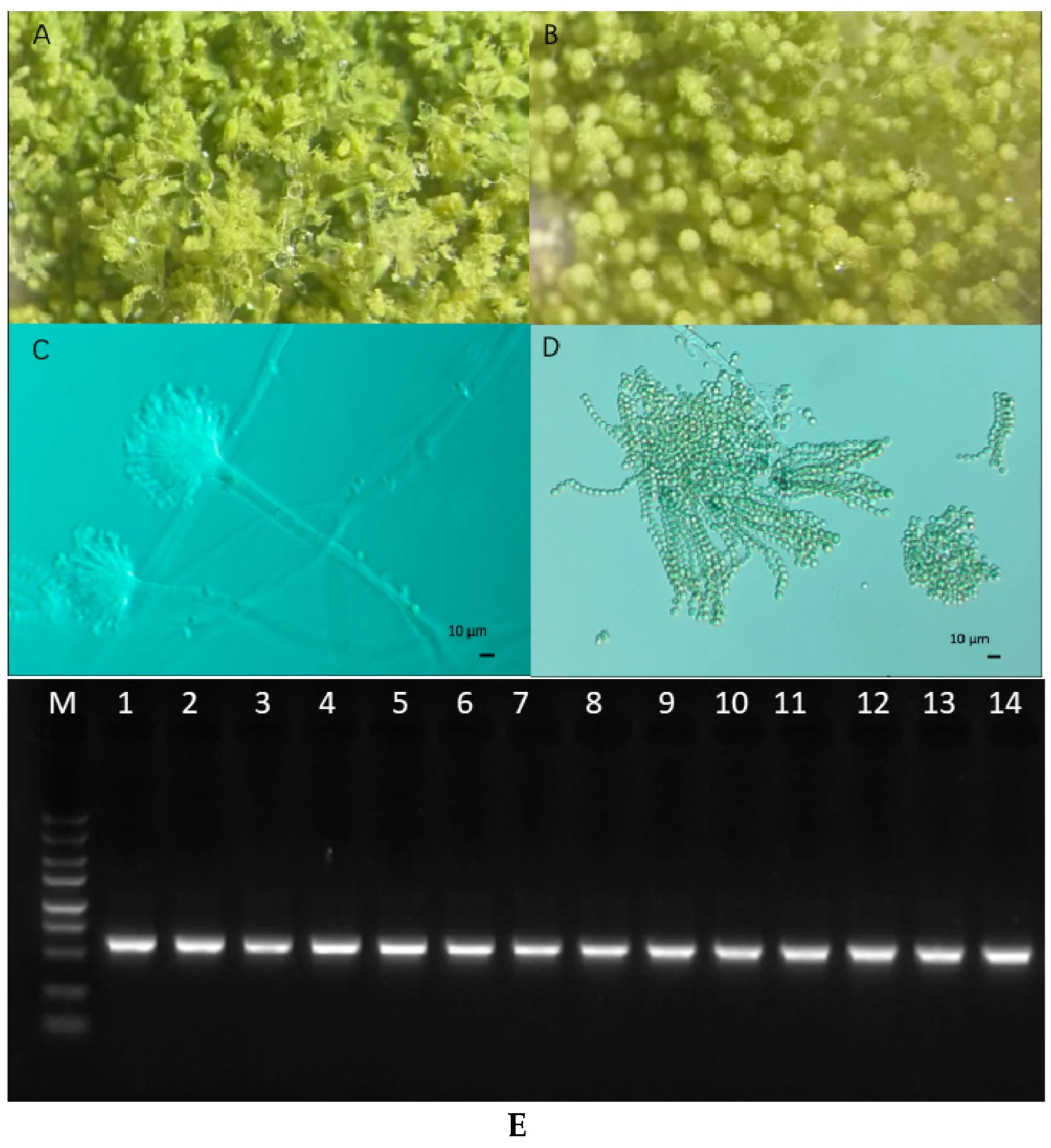
Morphological features and molecular confirmation of the *Aspergillus flavus* isolates. (**A**). Cylindrical conidial head; (**B**). spherical conidial head; (**C**). conidiogenous structure (400×); (**D**). conidia (400×). (**E**) shows the agarose gel electrophoresis bands of PCR-amplified DNA from 14 *A. flavus* isolates.

We isolated thirteen *A. flavus* isolates (HJ-1, HJ-2, HJ-4 to HJ-14) grown on selective media and then subjected them to morphological and molecular characterization. Microscopic analysis confirmed classical *A. flavus* characteristics, e.g., globose to subglobose conidial heads, biseriate phialides, and rough-walled conidia. Molecular identification by sequencing the ITS region showed 99–100% sequence similarity to reference strains of *A. flavus* in the NCBI database. These findings confirm that the selective media did not bias species recovery and facilitated accurate, reliable isolation of *A. flavus*. The microscopic examination of the selected *A. flavus* isolate is represented in Figure 4A–D, and gel electrophoresis of all isolates is given in Figure 4E. In contrast, the phylogenetic tree of the selected environmental and clinical isolates is given in Supplementary Figure S1.

To examine the biochemical basis of the observed increased growth and sporulation on peanut- and corn-based media and to evaluate whether these effects were common to both environmental and clinical isolates, we conducted high-resolution LC-MS/MS-based metabolomic profiling. We used selective media (PSM and CSM) and conventional media (PDA and SDA) to analyze environmental isolates of *Aspergillus flavus* (HJ-1 series) and a clinical isolate (L-1-22). Controls (peanut (CP) and corn (CC) media without *A. flavus* inoculum) were used to calculate background substrate-derived products.

For comparative metabolomics, one environmental strain, HJ-1, and one clinical isolate, L-1-22, were each cultivated on four media: peanut (HJ-1-P), Corn (HJ-1-C), PDA (HJ-1-PDA), and SDA (HJ-1-SDA clinical isolate L-1-22 was labeled as peanut (I), Corn (II), SDA (III), and PDA (IV). This design enables direct comparison of metabolic responses between media types and isolate origin. We assumed that the nutrient composition of the host-simulating media (peanut-based and corn-based media) would activate favorable metabolic reprogramming, and this mechanistic argument suggested that we would observe increased phenotypic performance and consistent selectivity in the isolated case.

### 3.5. Metabolomic Profiling of Aspergillus flavus Cultures

Non-targeted LC-MS/MS analysis of 10 samples, including both clinical and environmental *Aspergillus flavus* isolates cultivated on four different media and control media, provided strong, consistent statistical data. We detected 3635 metabolites across all samples, with 2872 detected with high confidence (MS/MS spectral match). High-quality control showed strong instrument stability and technical reproducibility. There was almost the same correlation in the QC samples (Pearson, r > 0.99), as well as low-intensity variance in internal standards (CV < 0.06), and over 75% of the detected metabolites had a QC CV of LS 0.3, which is a strong data stability measure (Supplementary Figure S2). This high data fidelity provided a solid baseline for the comparative analyses that followed.

#### 3.5.1. Global Metabolic Profiles Are Dictated by Strain Lineage and Growth Medium

**Figure 5:**
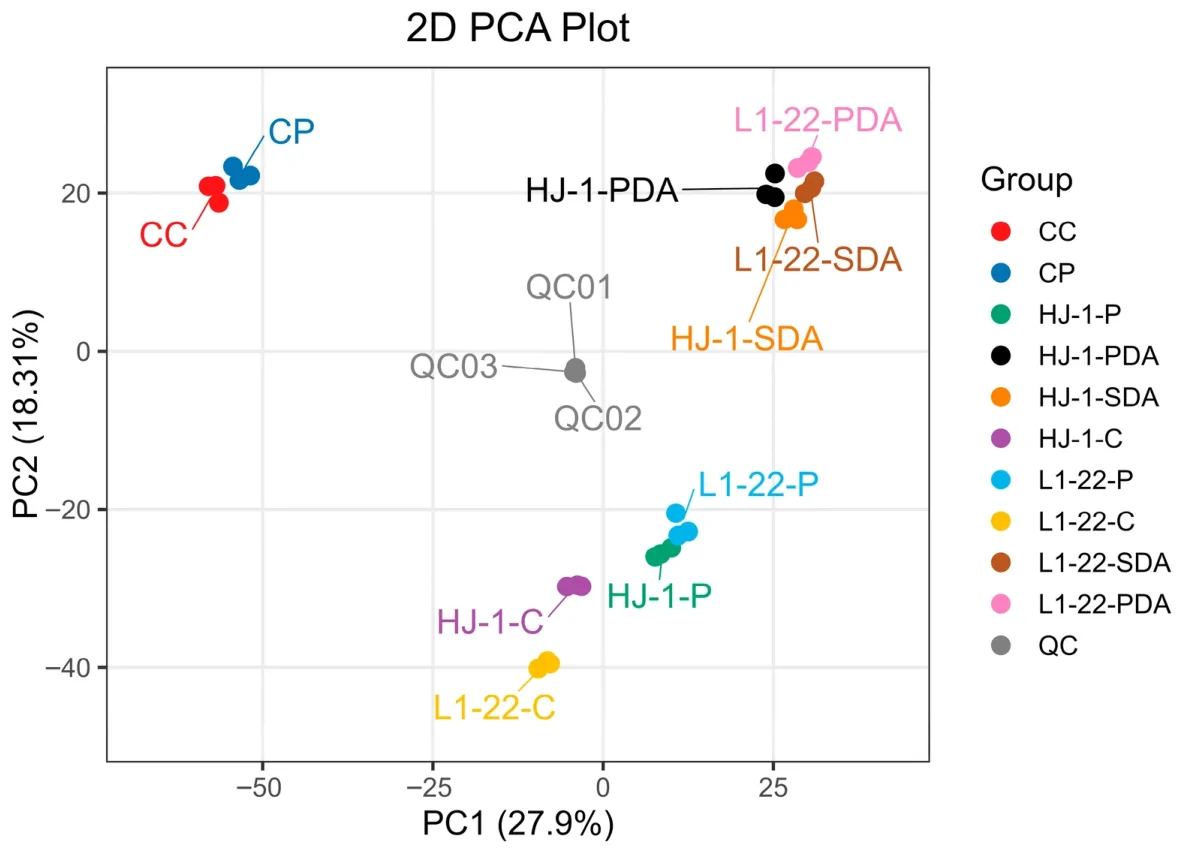
PCA scores plot of the metabolomic profiles of *Aspergillus flavus* grown on various culture media. The points are individual biological replicates (n = 3 per group); PC1 accounts for 27.9% of the variance, and PC2 accounts for 18.31% of the variance. Isolates cultivated on conventional media (PDA and SDA) clustered in the upper group, while isolates cultured on host-mimicking peanut (P) and corn (C) media clustered in the lower group, and uninoculated substrate controls (CP and CC) were distinctly separated from all fungal cultures. HJ-1, environmental isolate; L1-22, clinical isolate. The three quality-control (QC) samples clustered closely at the origin, indicating technical reproducibility and separation from all experimental samples, suggesting the observed variation was biological, not technical.

An unsupervised multivariate analysis showed significant metabolic differences, driven by both fungal strain and growth medium. All of these samples (n = 3 biological replicates per group) were grouped by medium type using principal component analysis (PCA), where the isolates grown in conventional media (PDA and SDA) were clustered in the upper region, the isolates grown in host-mimicking peanut and corn media were clustered in the lower region, and the uninoculated substrate controls (CP and CC) were clustered in the upper-left quadrant (Figure 5). PC1 separated the uninoculated controls and the clinical isolate L1-22-C from the other cultures but was less clear for the corn-grown L1-22-C. Interestingly, the clinical and environmental isolates grown on PDA and SDA were grouped together, while on the host-mimicking media they were clearly strain-specific separated (HJ-1-C vs. L1-22-C along PC1; HJ-1-P vs. L1-22-P mainly along PC2). All QC samples are tightly clustered at the origin, indicating technical reproducibility (Figure 5).

**Figure 6:**
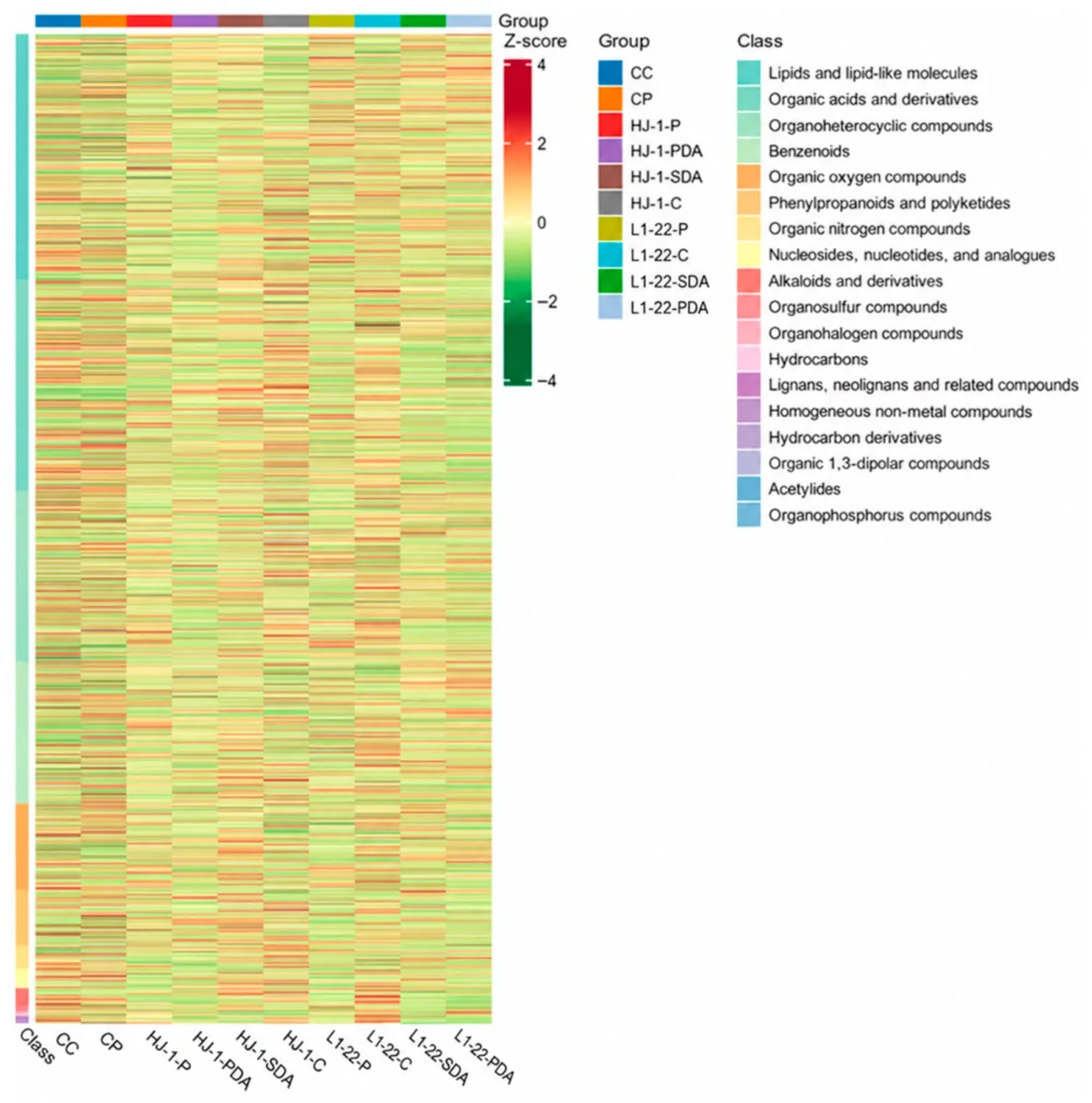
Hierarchical clustering heatmap of metabolite profiles across *Aspergillus flavus* isolates. Rows represent metabolite classes based on HMDB classification, while the columns represent isolates with their respective medium (CC: Corn Control; CP: Peanut Control; HJ-1-P: Environmental strain on Peanut; HJ-1-PDA: Environmental on PDA; HJ-1-SDA: Environmental on SDA; HJ-1-C: Environmental on Corn; L1-22-P: Clinical strain on Peanut; L1-22-C: Clinical on Corn; L1-22-SDA: Clinical on SDA; L1-22-PDA: Clinical on PDA). The color scale shows relative abundances (unit-variance scaling) of the metabolite classes, with red and green indicating high and low abundance, respectively. The dendrograms show similarity patterns in abundance profiles among metabolite classes (left) and samples (top). This analysis combines data from positive and negative ionization modes.

The control media (CC, CP) grouped distinctly from fungal cultures, validating that fungal metabolism rather than media drove the metabolome analyzed. Hierarchical clustering analysis supported this global structuring, where samples are mostly clustered based on strain origin and secondly on media type, forming a coherent cluster (Figure 6). Taken together, these analyses indicate that the clinical and environmental fitness of *A. flavus* reflect intrinsically distinct metabolic states that nutrient availability significantly remodels.

#### 3.5.2. Clinical and Environmental Strains Exhibit Widespread Metabolic Divergence

**Figure 7:**
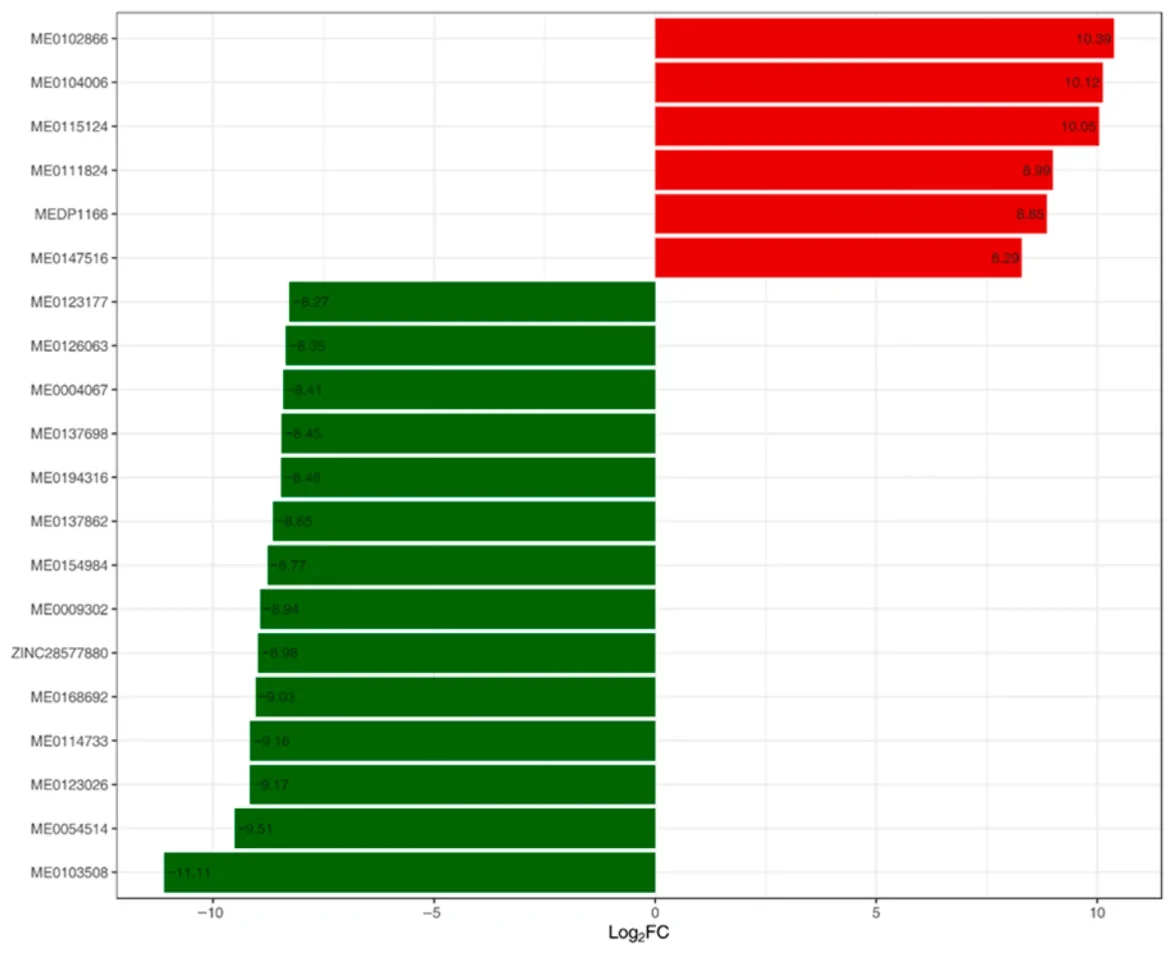
Clinical and environmental strains differ in metabolite abundance. The 20 highest-ranked metabolites by absolute Log2 fold change (clinical vs. environmental isolate) are presented. Red and green scales (or bars) denote upregulated and downregulated metabolites in the clinical strain, respectively.

To identify the metabolites underlying global separation, we performed pairwise differentiation analysis. Comparing clinical and environmental strains grown in similar media revealed a strong strain-specific effect. For instance, cultivation on a corn-based medium showed that the clinical isolate (II) expressed 125 metabolites with statistically significant changes in relative abundance compared with the environmental counterpart (HJ-1-C); of these, 57 were upregulated, and 68 were downregulated. A more focused analysis of the top twenty metabolites highlighted central biochemical discriminants, as presented in Figure 7.

Notably, the clinical strain showed marked upregulation of lipids such as lysophosphatidylcholine (LPC) (18:2_0:0) and the energy carrier acetyl-L-carnitine. In contrast, it downregulated several amino acids, such as tryptophan and arginine, agmatine (a neurotransmitter precursor), and nucleoside derivatives such as adenosine and 3-adenylic acid (Supplementary Table S8). A heatmap of all significant differential metabolites clearly shows consistent, diametrically opposite abundance patterns between the two strains in the media, supporting a basic metabolic reprogramming that supports the clinical phenotype (Supplementary Figure S3).

### 3.6. Nutrient Environment Significantly Reshapes the Fungal Metabolome

Growth medium had a considerable effect within strains. In the clinical isolate, the shift from peanut medium (I) to PDA (IV) modified 146 metabolites, whereas the shift from peanut medium (I) to SDA (III) modified 142 metabolites. We clustered all differential metabolites using k-means across all comparisons, producing six trends based on their abundance patterns across samples (Supplementary Figure S4). Some clusters showed high concentrations of metabolites abundant only in fungi grown on conventional media (PDA/SDA), indicating that rich, complex media strongly influence central metabolism. Other metabolite clusters, by contrast, were highly abundant only in PSM/CSM media.

#### 3.6.1. KEGG Pathway Enrichment Analysis of Metabolites

**Figure 8:**
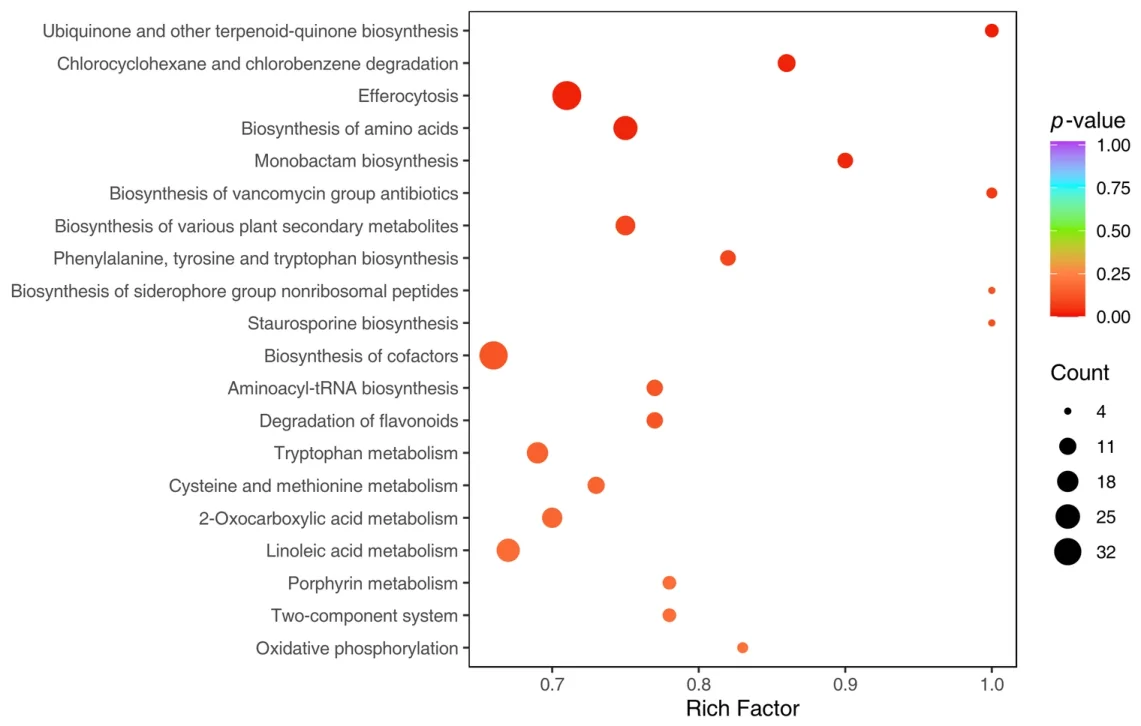
Pathway enrichment analysis of the metabolites that were differentially abundant among clinical and environmental *A. flavus* isolates using the KEGG database.

KEGG pathway enrichment analysis revealed pathways associated with amino acid biosynthesis and metabolism, secondary metabolite biosynthesis, cofactor biosynthesis, energy metabolism, and lipid-related processes. *p*-values and Benjamini–Hochberg FDR-adjusted *p*-values (Figure 8) were used to assess pathway enrichment. An FDR-adjusted *p*-value of <0.05 was considered significantly enriched. The enrichment analysis identified pathways including amino acid biosynthesis; phenylalanine, tyrosine, and tryptophan biosynthesis; tryptophan metabolism; biosynthesis of various secondary metabolites; cofactor biosynthesis; oxidative phosphorylation; and linoleic acid metabolism.

#### 3.6.2. Differential Abundance Analysis of Metabolic Pathways

**Figure 9:**
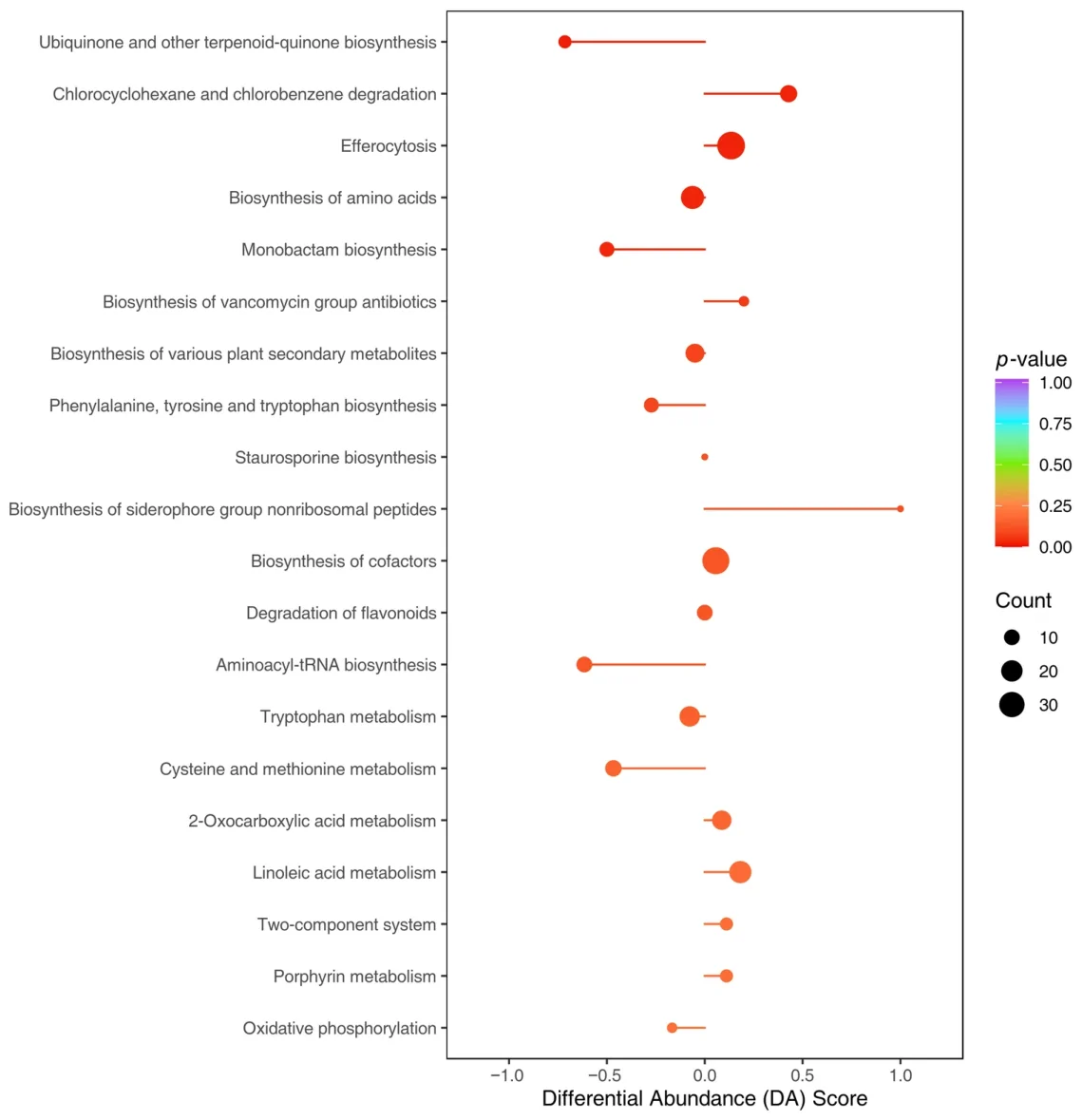
Metabolic pathway differential abundance (DA) score analysis of clinical and environmental *A. flavus* isolates.

To determine the overall direction of the change in these pathways, we calculated the Differential Abundance (DA) Score. This comparison revealed a systematic downregulation of amino acid metabolism pathways (e.g., phenylalanine, tyrosine, and tryptophan biosynthesis) in the clinical strain compared with the environmental strain (Figure 9). On the other hand, the pathways associated with glycerophospholipid metabolism were inclined to be upregulated. This coordinated response suggests that, under these experimental conditions, the clinical strain promotes lipid remodeling and peripheral biosynthesis at the expense of maintaining free amino acid pools.

## 4. Discussion

This study introduces two key contributions: a practical, effective tool for isolating *Aspergillus flavus* and new insight into the metabolic factors that drive strain differentiation, both grounded in ecological relevance [35,36]. Moving beyond traditional, nutrient-rich media, our work highlights how the substrate itself serves as a critical variable that can uncover the true ecological behavior of *A. flavus* [37].

### 4.1. A Methodological Paradigm Shift: From Enrichment to Ecological Simulation

Traditional media such as PDA and SDA are commonly used for broad fungal enrichment. However, they lack specificity and do not accurately reflect the ecological conditions that fungi experience in nature [38]. These media, often high in carbohydrates, create artificial environments that hinder selective isolation and can obscure important ecological traits [39].

In contrast, our innovative approach uses Peanut-Based Selective Medium (PSM) and Corn-Based Selective Medium (CSM), which mimic the natural substrates that *A. flavus* encounters in its environment [40]. By adding selective agents and adjusting physicochemical conditions, we created an environment that selects for *A. flavus* while also providing the nutrients it would naturally encounter [41].

This has dramatically reduced background microbiota, increased yields of near-pure cultures, and significantly improved both radial growth and conidiation [42,43]. These results validate the approach and establish a new paradigm in which “host-mimicking” media connect field ecology with laboratory research, yielding more accurate in vitro findings [44].

### 4.2. Metabolomic Reprogramming of Clinical A. flavus

Non-targeted metabolomic profiling reveals biological variation not captured by growth alone [45]. Although cultivated under the same conditions, clinical and environmental *A. flavus* strains formed distinct clusters in PCA, suggesting inherently different metabolic states rather than media-induced differences [46]. This divergence in clinical strains is characterized by general inhibition of central amino acid biosynthesis pathways (tryptophan and arginine) and a substantial increase in glycerophospholipid metabolism [47,48]. This metabolic profile aligns with an adaptive allocation of resources shaped by the host environment.

Within a host, clinical isolates likely had access to ready pools of amino acids, making pressure to preserve energetically costly de novo synthesis routes less important. Similar dependence on host-derived nutrients has been common in reports of fungal pathogens evolving to host-associated lifestyles [49]. At the same time, selective investment in lipid metabolism seems particularly interesting. Glycerophospholipids and host-derived lipids are membrane components and are already known to participate in stress responses, signaling cascades, and virulence in *Aspergillus* spp. [50,51,52].

Additional evidence for this interpretation includes enrichment of metabolites such as lysophosphatidylcholine, linked to membrane remodeling and stress adaptation [48], and acetyl-L-carnitine, a central actor in mitochondrial lipid-based energy production during stress [53]. Taken together, these metabolic characteristics indicate a plastic, lipid-converged physiological state that can improve adaptation to host-enforced oxidative stress and alter responses to antifungal therapy. This condition may represent metabolic pre-adaptation that favors persistence and pathogenicity in mammalian hosts, consistent with broader studies of fungal evolution in chronic infection environments [54].

The clinical metabolic phenotype mentioned above was found only in a single clinical isolate, L-1-22. There is a well-documented intraspecific metabolic diversity of *A. flavus* isolates, and the divergence of L-1-22 from the environmental isolate could be reflective of idiosyncrasies at the level of isolates (host-adaptation history, geographic origin, or genotype) and not a universal signature of clinical *A. flavus.* These results are therefore an exploratory, proof-of-concept comparison that suggests testable hypotheses (e.g., the lipid-versus-amino-acid trade-off), rather than a general model for the metabolism of clinical *A. flavus.* The boundaries of the clinical metabolic phenotype will be set by metabolomic profiling of a larger geographically and clinically diverse panel of clinical isolates, preferably run in parallel to matched environmental strains from the same area.

### 4.3. Nutrient as a Directive Signal: Medium-Dependent Metabolic Rewiring

The study also highlights the strong effect of the growth medium. Hundreds of metabolites change when researchers replace potato dextrose agar (PDA) with peanut-based specialized media (PSM), reflecting the high plasticity of fungal metabolism [51,55]. This substrate-specific reprogramming has implications for understanding how secondary metabolism is regulated, including mycotoxin biosynthesis pathways [56,57]. The host-mimicking (PSM) medium is a promising platform for future studies on mycotoxin biosynthesis regulation, since secondary metabolite production is well known to be sensitive to nutrients [58,59,60].

## 5. Conclusions

This study goes beyond optimizing colony counts. It shows that the culture medium is a crucial experimental variable that can either reveal or obscure the true biology of *A. flavus*. We developed a better isolation medium that recapitulates key features of the natural host and elucidated the central metabolic schism between clinical and environmental *A. flavus*. This work provides a strong framework and a powerful toolkit for future studies aimed at understanding and coping with the threats posed by this versatile pathogen.

## 6. Limitations and Future Perspectives

Although our metabolomic results are correlative, they indicate strong functional adaptations. A critical limitation of this study is that the clinical metabolic phenotype was characterized using only a single clinical isolate (L-1-22), and therefore no generalization should be drawn to the broader population of clinical *A. flavus*. The differences in amino acid and lipid metabolism observed may reflect isolate-specific characteristics rather than a conserved signature of clinical disease, and we have not assessed intra-specific differences among clinical isolates.

Future research should incorporate transcriptomics to link these metabolic changes to regulatory gene networks and to inform and predict the role of specific lipid pathways in virulence via genetic knockouts, since these differences may not reflect a conserved signature of clinical disease. Future studies should also include a larger, more geographically representative sample of clinical and environmental isolates to define the prevalence and variability of the clinical metabolic phenotype. The applicability of the PSM/CSM to a worldwide strain collection must be tested by expanding it to a global strain collection. Finally, direct comparisons of virulence properties (e.g., macrophage survival, murine models) and secondary metabolite production (aflatoxins) in strains pre-adapted on PSM or PDA would definitively confirm the physiological relevance of our host-mimicking platform. There was no quantitative measurement of aflatoxins. PSM/CSM formulation was not optimized empirically, but determined a priori. In conclusion, all the metabolic results reported here are hypothesis-driven for these clinical strains and need to be verified in larger collections of strains.

## Data Availability

The raw data supporting the conclusions of this article will be made available by the authors on request.

## Supplementary Materials

The following supporting information can be downloaded at https://www.mdpi.com/article/10.3390/microorganisms14102391/s1. Table S1. Geographic distribution of sampling sites for *Aspergillus flavus*. Table S2. Primers used for each respective gene of *A. flavus*. Table S3. Isolates, location and gene accession number. Table S4. Mobile Phase Gradient Program for HSS T3 Column Separation. Table S5. Detailed Q Exactive HF-X mass spectrometry acquisition parameters. Table S6. The % recovery of *A. flavus* on novel PSM and CSM media with conventional PDA and SDA media. Table S7 colony diameter of *A. flavus* on four different media. Table S8. Metabolic reprogramming in a clinical *Aspergillus flavus* isolate. Figure S1. Phylogenetic tree of *Aspergillus flavus* based on ITS sequence. Figure S2. Quality control of the non-targeted metabolomic data. Figure S3. Coordinated change in metabolic classes. Figure S4. The K-means clustering of differential metabolites shows six abundance patterns between media and strains.
