# Supplementary material for: A Host-Mimicking Peanut-Based Selective Medium Enhances Isolation, Sporulation, and Metabolomic Profiling of Aspergillus flavus
Source: Microorganisms. 2026 Oct 19;14(10):2391. doi: 10.3390/microorganisms14102391 (PMC13707809; doi:10.3390/microorganisms14102391)
Supplement: Supplementary file 1 [file microorganisms-14-02391-s001.zip › microorganisms-4611926-supplementary.pdf]

A Host-Mimicking Peanut-Based Selective Medium Enhances Isolation, Sporulation, and Metabolomic Profiling of *Aspergillus flavus*

Supplementary data

Table S1. Geographic distribution of sampling sites for Aspergillus flavus. We isolated 14 strains (HJ-1 to HJ-14) from environmental substrates in Guizhou and Xinjiang province and a clinical isolate L-1-22 from respiratory organs in Jining, SD Province, China.

Sample ID	Original Location (China)	Latitude (N)	Longitude (E)	Elevation
HJ-1	Huaxi District, Guizhou (Corn)	26.4117°	106.6713°	~1,120 m
HJ-2	Tongren, Guizhou (peanut)	27.7319°	109.1894°	~400 m
HJ4-HJ7	Changji, Xinjiang (cornfield soil)	44.0230°	87.3123°	~580 m
HJ8-HJ10	Xiuwen County, Guizhou (peanut & corn)	26.8416°	106.5942°	~1,200 m
HJ11, 12	Shiqian County, Guizhou (peanut & beans)	27.5180°	108.2294°	~450 m
HJ-13, 14	Renhuai City, Zunyi, Guizhou (fermented materials)	27.8662°	106.3785°	~400 m
L-1-22	Jining, Shandong (clinical respiratory specimen)	35.4152°	116.5872°	~40 m

Table S2. Primers used for each respective gene of A. flavus

Locus	Primer name	Direction	Sequence (5′→3′)
β-tubulin (BenA)	(β-tub1)	Forward	AATTGGTGCCGCTTTCTGG
	(β-tub2)	Reverse	AGTTGTCGGGACGGAATAG
Calmodulin (CaM)	CMD5	Forward	CCGAGTACAAGGARGCCTTTC
	CMD6	Reverse	CCGATRGAGGTCATRACGTGG
Internal transcribed spacer (ITS)	ITS1	Forward	TCCGTAGGTGAACCTGCGG
	ITS4	Reverse	TCCTCCGCTTATTGATATGC

Table S3. Isolates, location and gene accession number

Sample ID	Original Location (China)	GenBank Accession No		
		ITS	BenA	CaM
HJ-1	Huaxi District, Guizhou (Corn)	PZ747462	PZ766678	PZ766968
HJ-2	Tongren, Guizhou (peanut)	-	-	-
HJ4	Changji, Xinjiang (cornfield soil)	PZ747464	PZ766680	PZ766970
HJ5	Changji, Xinjiang (cornfield soil)	PZ747465	PZ766681	PZ766971
HJ6	Changji, Xinjiang (cornfield soil)	PZ747466	PZ766682	PZ766972
HJ7	Changji, Xinjiang (cornfield soil)	PZ747467	PZ766683	PZ766973
HJ8	Xiuwen County, Guizhou (peanut & corn)	PZ747468	PZ766684	PZ766974
HJ9	Xiuwen County, Guizhou (peanut & corn)	PZ747469	PZ766685	PZ766975
HJ10	Xiuwen County, Guizhou (peanut & corn)	PZ747470	PZ766686	PZ766976
HJ11	Shiqian County, Guizhou (peanut & beans)	PZ747471	PZ766687	PZ766977
HJ12	Shiqian County, Guizhou (peanut & beans)	PZ747472	PZ766688	PZ766978
HJ13	Renhuai City, Zunyi, Guizhou (fermented materials)	PZ747473	PZ766689	PZ766979
HJ14	Renhuai City, Zunyi, Guizhou (fermented materials)	PZ747474	PZ766690	PZ766980
L-1-22	Jining, Shandong (clinical respiratory specimen)	PZ747462	PZ766678	PZ766968

Table S4 mobile Phase Gradient Program for HSS T3 Column Separation.

**Time (min)**	**A (%)**	**B (%)**
0	95	5
1	80	20
3	1	99
4.5	1	99
4.6	95	5
6	95	5
Note: Mobile phase A: 0.1% formic acid in water; Mobile phase B: 0.1% formic acid in acetonitrile.		

Table S5. Detailed Q Exactive HF-X mass spectrometry acquisition parameters.

Parameter	**ESI+**	**ESI-**
Spray Voltage (V)	3800	3400
Sheath Gas Flow (Arb)	60	60
Auxiliary Gas Flow (Arb)	20	20
Ion Transfer Tube Temperature (°C)	320	320
Vaporizer Temperature (°C)	350	650
Full MS (MS^1) Scan		
Scan Range (*m/z*)	70-1000	70-1000
Resolution	60,000	60,000
AGC Target	3.00E+06	3.00E+06
Data-Dependent MS/MS (MS^2) Scan		
Scan Range (*m/z*)	70-1000	70-1000
Resolution	15,000	15,000
AGC Target	1.00E+05	1.00E+05
RF Lens (%)	50	50
Stepped Normalized Collision Energy (NCE)	30, 40, 50	30, 40, 50
Data-Dependent Acquisition (DDA) Settings		
Signal Intensity Threshold (cps)	1.00E+06	1.00E+06
TopN	10	10
Dynamic Exclusion Window (s)	3	3
Note: Arb, arbitrary units; AGC, automatic gain control; NCE, normalized collision energy; cps, counts per second.		

Table S6. The % recovery of A. flavus on novel PSM and CSM media with conventional PDA and SDA media

**Media**	**Total colonies**	***A. flavus* colonies**	**Recovery %**	**Selectivity**
PDA	14	13	85.71	Low
SDA	11	9	81.81	Low
PSM	2	2	100	High
CSM	3	3	100	High

Table S7 colony diameter of A. flavus on four different media.

Index	Compounds	Type
ME0153243	LPC (18:2_0:0)	up
ME0193946	Tryptophan	down
ME0194064	Acetyl-L-carnitine	up
ME0159989	Arginine	down
ME0110897	Agmatine	up
MEDP0125	Choline	down
ME0168692	Adenosine	down
MEDP0513	Palmitic amide	down
ME0003626	3,4-Dihydroxymandelic acid	up
MEDL02151	3’-Adenylic acid	down

Table S8. Metabolic reprogramming in a clinical Aspergillus flavus isolate. Selected metabolites that were significantly upregulated or downregulated in the clinical strain compared to the environmental strain when cultivated on the same medium. The pattern highlights concurrent

Strain Number	CSM	PSM	PDA	SDA
	Colony Diameter (cm)			
1	7.63±0.15	7.87±0	6.57±0.12	6.63±0.12
2	7.60±0.10	8.27±0.06	5.77±0.15	6.80±0
3	6.73±0.06	7.57±0.21	6.50±0.17	6.57±0.15
4	7.93±0.12	8.00±0	5.5±0.17	6.80±0.10
5	6.77±0.06	7.40±0.17	5.40±0.10	6.47±0.06
6	7.73±0.06	8.27±0.06	5.37±0.06	6.83±0.06
7	7.37±0.06	8.00±0	5.53±0.06	6.63±0.06
8	7.13±0.15	7.63±0.15	5.30±0.10	6.37±0.15
9	7.10±0.10	7.83±0.06	5.63±0.06	6.50±0.10
10	7.90±0.10	8.00±0	5.37±0.06	6.73±0.06
11	7.60±0.10	8.00±0	5.47±0.06	6.77±0.06
12	7.50±0.10	7.93±0.12	5.40±0.10	7.00±0.10
13	7.57±0.06	8.23±0.06	5.17±0.15	5.73±0.06
14	7.40±0.10	8.20±0.10	5.23±0.06	6.70±0.17

shifts in lipid, amino acid, energy, and nucleotide metabolism.

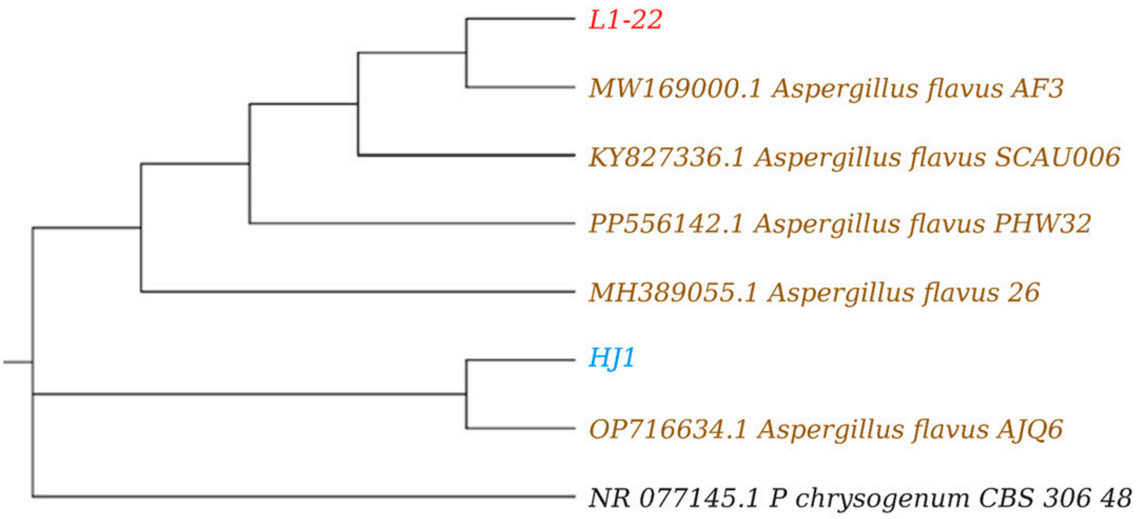

Figure S1. Phylogenetic tree of Aspergillus flavus based on ITS sequence. The clinical isolate (Aspergillus flavus-1 clinical) is colored red, the environmental isolate (HJ1 environmental) is colored blue, and the reference strains are indicated in gray. The Fungus used were A. flavus strains accessed from GenBank, denoted in italics. Penicillium chrysogenum CBS 306.48 was used as an outgroup to root the tree. Sequence divergence is proportional to the length of the branch.

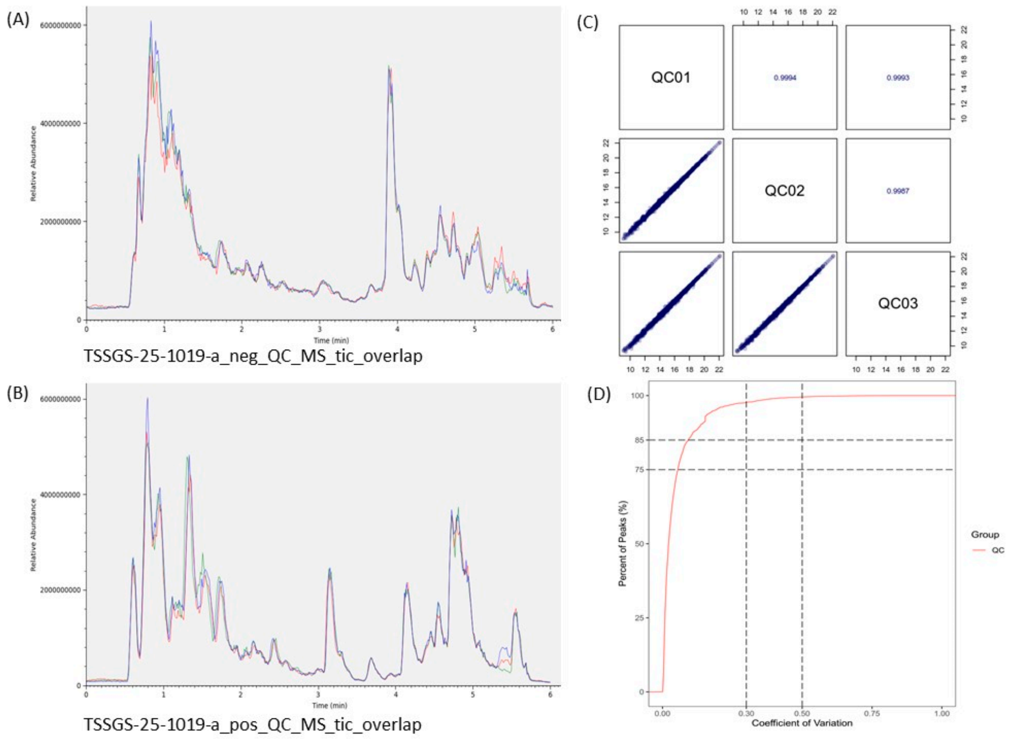

Figure S2. Quality control of the non-targeted metabolomics data. (A, B) Total ion chromatograms (TICs) of all quality control (QC) samples obtained in both negative and positive ionization conditions, respectively; (C) Correlation of the QC samples. The upper triangle displays Pearson correlation coefficients (r) for all pairwise comparisons; the lower triangle displays the corresponding scatter plots of log-transformed metabolite abundances. (D) Cumulative distribution function (ECDF) plot of the coefficient of variation (CV) of all the groups of samples. The dashed vertical lines show CV thresholds of 0.3 and 0.5, and the horizontal line shows the 75% and 85% quantiles. The CV of more than 75% of the metabolites in QC samples was below 0.3, which also indicates excellent analysis precision and stability in the dataset

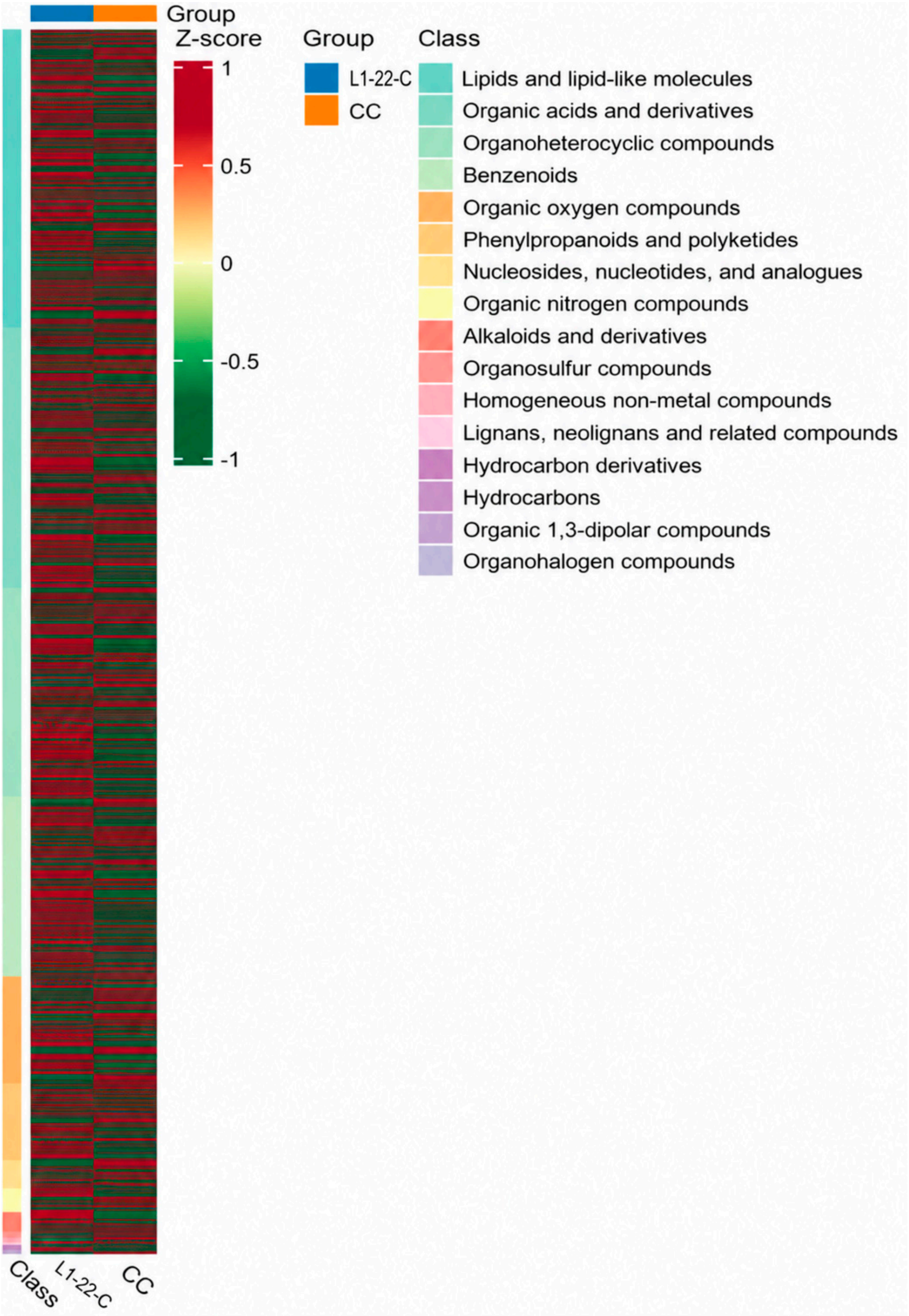

Figure S3. Co-ordinated change in metabolic classes. Z-score analysis to indicate phenotype-dependent metabolic reprogramming showing the converse abundance pattern of key biochemical classes between clinical and environmental fungal strains.

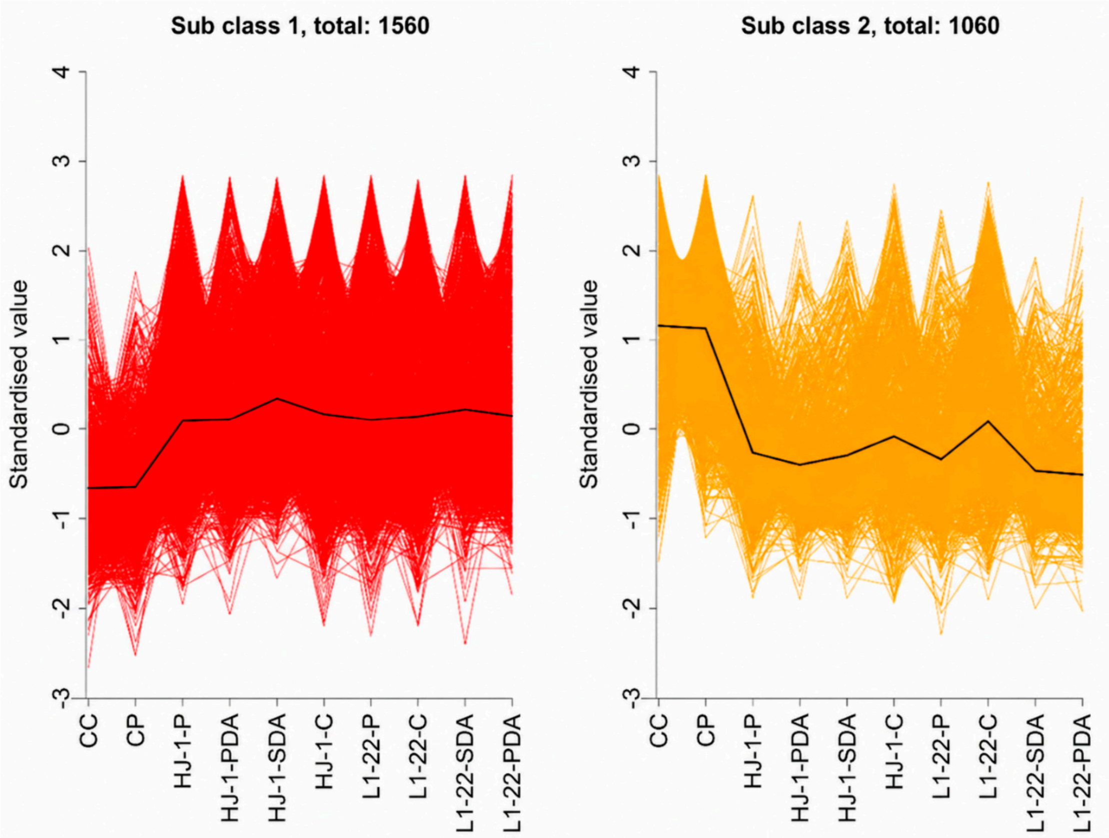

Figure S4. The k-means clustering of differential metabolites demonstrates six abundance patterns between media and strains. The heatmap represents the standardized abundance (z-scores) of all differentially abundant metabolites clustered in six clusters with the help of the k-means method. The rows and columns represent the metabolites and the various growth media and the different strains (CC, CP, HJ-1 variants, clinical isolates, L1-22). The color gradient captures relative abundance of metabolites to illustrate the changes in the metabolite profile with medium composition as well as in relation to clinical backgrounds and environmental backgrounds. This coordinated metabolic reprogramming is emphasized by this clustering and is caused by both genetic origin and contextual environmental factors.
